# Commentary: The Nature of Unsymbolized Thinking

**DOI:** 10.3389/fpsyg.2018.00216

**Published:** 2018-02-22

**Authors:** Daniel Gregory

**Affiliations:** Department of Philosophy, University of Fribourg, Fribourg, Switzerland

**Keywords:** unsymbolized thinking, unsymbolized thought, inner speech, internal speech, descriptive experience sampling

Vicente and Martínez-Manrique's paper deserves attention because it offers an attractive explanation of a phenomenon which, if real, is intriguing. The phenomenon is “unsymbolized thinking” (UT): “thinking a particular, definite thought without the awareness of that thought's being conveyed in words, images, or any other symbols” (Hurlburt and Heavey, 2008, p. 802). After reviewing their explanation, I want to raise a question about it.

Descriptive Experience Sampling is a method for investigating mental experience developed by Russell Hurlburt and various colleagues. Subjects wear devices which beep at random times as they carry out their daily routines. When the device beeps, a subject records what was happening in their mind immediately beforehand. Researchers then meet with the subjects and discuss the experiences they have noted, to help them describe those experiences faithfully. The process is repeated typically four to eight times (e.g., Hurlburt and Akhter, 2006 for more detail). Allegedly, one kind of experience subjects report is UT (e.g., Hurlburt and Akhter, 2008; Hurlburt and Heavey, 2008). Here is an example:

Benito is watching two men carry a load of bricks in a construction site. He is wondering whether the men will drop the bricks. This wondering does not involve any symbols, but it is understood to be an explicit cognitive process (Hurlburt and Akhter, 2008, p 1364).

Vicente and Martínez-Manrique briefly discuss concerns that have been raised about DES. However, they “prefer to take the phenomenon [of UT] at face value” and their project is to explain it (p. 174). They allow, though, that “the unconvinced reader can take [their] task as conditional: if there is [such] a … phenomenon, what explanation could it have?” (p. 174). Their explanation draws on a common account of inner speech (they cite Carruthers, 2011, Swiney and Sousa, 2014), and proceeds as follows.

When we produce motor commands, a mechanism in the brain predicts the proprioceptive and sensory feedback we will receive when we perform the motor commands. If you, say, move your hand in front of your face, this mechanism predicts that you will have the proprioceptive sensation of your hand so moving and that you will see a hand move across your visual field. A match between predicted and actual feedback is what underlies a subject's sense of agency in performing a physical action. Vicente and Martínez-Manrique mention Helmholtz (1860), von Holst and Mittelstaedt (1950), and Sperry (1950) as historical sources.

It has been suggested that mental imagery occurs when a motor command is issued, and so a prediction is produced about the feedback one would receive if the command were executed, but for some reason it is not carried out (Vicente and Martínez-Manrique cite Jeannerod, 2006, Guillot et al., 2012). Supposing this is right, “[a]n episode of inner speech … could be a prediction issued on the basis of an aborted motor command for [external] speech production, a prediction about the incoming sensory signal that the subject would produce if he/she had executed the motor command” (p. 180).

But Vicente and Martínez-Manrique believe that something happens before this when we produce inner speech. For them, we first form an intention to express a particular meaning. A prediction is issued at this stage as well: a prediction that the meaning will somehow be expressed. We then formulate the phrasing we will use and issue the motor commands to produce the utterance externally; an utterance in inner speech is produced if the motor commands are aborted. Accordingly, the production of inner speech involves two predictions: one about the expression of a meaning; one about the sound of an utterance expressing it (they cite Pickering and Garrod, 2013). Vicente and Martínez-Manrique note Jeannerod's (1995) suggestion that “predictions are made conscious just by being predictions of aborted actions, that is, if an action is aborted after the prediction is issued, the prediction will make it into consciousness” (p. 180; Vicente and Martínez-Manrique's wording). Vicente and Martínez-Manrique's innovative proposal is that, if the process is aborted at the early stage—if the intention to express a particular meaning is abandoned—then what results is a UT. One has a conscious experience of the meaning, but not of any representation of it.

The question that gives me pause is this: what is predicted at the first stage, when the intention to express a particular meaning is formed? Vicente and Martínez-Manrique write that “what we predict is that a certain thought-content, which uses the semantics of our language, is expressed. The content of the prediction is precisely the thought-content” (pp. 180–181). Now, the only type of thing that can be predicted is an event; a prediction is a forecast of something happening. It makes sense to speak of predicting the expression of a thought-content; the expression of a thought-content is an event. But it does not make sense to say that “[t]he content of the prediction is precisely the thought-content.” Thought-content itself is not an event that can be predicted.

Can we simply say that it is in fact the expression of a thought-content which is predicted, not the thought-content itself? No: if the expression of a thought-content were predicted and then abandoned, what becomes conscious should still be the representation of some kind of *event* of expression. But there is no suggestion that UTs involve the conscious representation of events of expression. UTs are supposed to involve something like the bare experience of thought-contents. And a bare experience of a thought-content is not a representation of an event of expressing that thought-content. (Though, of course, an event of expression can involve a representation of thought-contents).

There is something about Vicente and Martínez-Manrique's proposal that rings true: there could well be a connection between inner speech and UT. In order for the suggestion to be properly evaluated, though, I think this point needs to be clarified.

(Note: Vicente and Jorba (forthcoming) elaborates the proposal, but does not address the issue I raise here. See Martínez-Manrique and Vicente (2015) for an earlier statement of their view).

## Author contributions

The author confirms being the sole contributor of this work and approved it for publication.

### Conflict of interest statement

The author declares that the research was conducted in the absence of any commercial or financial relationships that could be construed as a potential conflict of interest.
